# Relationship between location and size of myocardial infarction and their reciprocal influences on post-infarction left ventricular remodeling

**DOI:** 10.1186/1532-429X-13-S1-P84

**Published:** 2011-02-02

**Authors:** Pier Giorgio Masci, Javier Ganame, Marco Francone, Walter Desmet, Valentina Lorenzoni, Ilaria Iacucci, Andrea Barison, Iacopo Carbone, Luciano Agati, Massimo Lombardi, Stefan Janssens, Jan Bogaert

**Affiliations:** 1 Fondazione Toscana/CNR "G. Monasterio", Pisa, Italy; 2 Gasthuisberg University Hospital, Leuven, Belgium; 3 La Sapienza University, Rome, Italy; 4 Institute of Clinical Physiology/CNR, Pisa, Italy; 5 Scuola Superiore S.Anna, Pisa, Italy

## Background

Patients with anterior MI experience worse LV remodeling and dysfunction than non-anterior MI patients. It remains unclear whether this difference is due to larger MI size or whether infarct location plays a role beyond MI size.

## Study aim

To assess the relationship between myocardial infarction (MI) location and size and their reciprocal influences on post-infarction left ventricular (LV) remodeling.

## Methods

A cohort of 260 reperfused ST-segment elevation MI patients was studied with cardiovascular magnetic resonance (CMR) at 1-week (baseline) and 4-month (follow-up). Area at risk (AAR) and MI size were quantified by T2-weighted and late gadolinium enhancement imaging, respectively. Adverse LV remodeling was defined as increase in LV end-systolic volume ≥15% at follow-up.

## Results

One-hundred twenty-seven(49%) patients had anterior MI and 133(51%) patients had non-anterior MI. Although the degree of myocardial salvage was similar between groups (p=0.74), anterior MI patients had larger AAR and MI size than non-anterior MI patients yielding worse regional and global LV function at baseline and follow-up. At univariable analysis, anterior MI was associated with increased risk of adverse LV remodeling (p=0.017) and lower LV ejection-fraction at follow-up (p=0.001), but not when accounted for baseline MI size. Accordingly, at multivariable analysis baseline MI size but not its location was an independent predictor of adverse LV remodeling (OR=1.061, p<0.001) and ejection-fraction at follow-up (Beta-coefficient=-0.255, p<0.001).

## Conclusions

Anterior MI patients experience more pronounced post-infarction LV remodeling and dysfunction than non-anterior MI patients due to greater magnitude of irreversible ischemic LV damage without any independent contribution of MI location.

